# Preparation and Characterization of Avenin-Enriched Oat Protein by Chill Precipitation for Feeding Trials in Celiac Disease

**DOI:** 10.3389/fnut.2019.00162

**Published:** 2019-10-15

**Authors:** Greg Tanner, Angéla Juhász, Christakis George Florides, Mitchell Nye-Wood, Frank Békés, Michelle L. Colgrave, Amy K. Russell, Melinda Y. Hardy, Jason A. Tye-Din

**Affiliations:** ^1^School of Biosciences, University of Melbourne, Melbourne, VIC, Australia; ^2^School of Science, Edith Cowan University, Joondalup, WA, Australia; ^3^School of Veterinary and Life Sciences, Murdoch University, Murdoch, WA, Australia; ^4^FBFD PTY LTD, Sydney, NSW, Australia; ^5^Immunology Division, The Walter and Eliza Hall Institute, Melbourne, VIC, Australia; ^6^Department of Medical Biology, The University of Melbourne, Melbourne, VIC, Australia; ^7^Department of Gastroenterology, The Royal Melbourne Hospital, Parkville, VIC, Australia; ^8^Murdoch Children's Research Institute, Royal Children's Hospital, Melbourne, VIC, Australia

**Keywords:** oats, avenin, gluten-free diet, LC-MS, MALDI-TOF, celiac disease

## Abstract

The safety of oats for people with celiac disease remains unresolved. While oats have attractive nutritional properties that can improve the quality and palatability of the restrictive, low fiber gluten-free diet, rigorous feeding studies to address their safety in celiac disease are needed. Assessing the oat prolamin proteins (avenins) in isolation and controlling for gluten contamination and other oat components such as fiber that can cause non-specific effects and symptoms is crucial. Further, the avenin should contain all reported immunogenic T cell epitopes, and be deliverable at a dose that enables biological responses to be correlated with clinical effects. To date, isolation of a purified food-grade avenin in sufficient quantities for feeding studies has not been feasible. Here, we report a new gluten isolation technique that enabled 2 kg of avenin to be extracted from 400 kg of wheat-free oats under rigorous gluten-free and food grade conditions. The extract consisted of 85% protein of which 96% of the protein was avenin. The concentration of starch (1.8% dry weight), β-glucan (0.2% dry weight), and free sugars (1.8% dry weight) were all low in the final avenin preparation. Other sugars including oligosaccharides, small fructans, and other complex sugars were also low at 2.8% dry weight. Liquid chromatography tandem mass spectrometry (LC-MS/MS) analysis of the proteins in these preparations showed they consisted only of oat proteins and were uncontaminated by gluten containing cereals including wheat, barley or rye. Proteomic analysis of the avenin enriched samples detected more avenin subtypes and fewer other proteins compared to samples obtained using other extraction procedures. The identified proteins represented five main groups, four containing known immune-stimulatory avenin peptides. All five groups were identified in the 50% (v/v) ethanol extract however the group harboring the epitope DQ2.5-ave-1b was less represented. The avenin-enriched protein fractions were quantitatively collected by reversed phase HPLC and analyzed by MALDI-TOF mass spectrometry. Three reverse phase HPLC peaks, representing ~40% of the protein content, were enriched in proteins containing DQ2.5-ave-1a epitope. The resultant high quality avenin will facilitate controlled and definitive feeding studies to establish the safety of oat consumption by people with celiac disease.

## Introduction

Establishing a safe gluten-free diet (GFD) for people with celiac disease (CD) is important for the health of the 1.4% of affected sufferers globally (1). CD is a chronic immune illness with features closely related to autoimmunity, and its pathogenesis is strongly linked to CD4^+^ T cells that are activated by dietary gluten peptides (2). Since the 1950s, after gluten was identified as the causative antigenic trigger, its sole treatment has been strict and lifelong gluten exclusion by removing wheat, barley, and rye. In this clinical context, the term gluten encompasses all of the pathogenic prolamin proteins from wheat (gliadin and glutenin), barley (hordein) and rye (secalin) that are immunotoxic in CD.

Oats contain a gluten-like prolamin protein called avenin and early food challenge studies indicated they can cause clinical relapse in some people with CD (3). More recent oat feeding studies that control for the confounding effect of wheat and barley contamination have indicated that pure oats are safe in CD although methodological limitations of these studies have been identified (4). A few clinical studies have shown mucosal inflammation (5), raised intra-epithelial lymphocytes (6, 7), or villous atrophy (8) induced by oats, suggestive of an adverse CD effect. A variety of *in vitro* studies have shown oat fractions can induce pro-inflammatory immune effects (9–11). Importantly, key avenin peptides that stimulate the pathogenic gluten-specific T cells in CD patients *in vivo* have been defined (12, 13). These peptides contain the immunodominant T cell epitopes DQ2.5-ave-1a (PYPEQEEPF), DQ2.5-ave-1b (PYPEQEQPF), DQ2.5-ave-1c (PYPEQEQPI), and DQ2.5-ave-2 (PYPEQQPF) with close sequence homology to barley T cell epitopes immunotoxic in CD such as DQ2.5-hor-3 (PIPEQPQPY) (14). The collective uncertainty of these findings regarding the true clinical safety of oats in CD has translated into different feeding recommendations; while Australia and New Zealand mandate the exclusion of oats from the GFD, most countries do not.

Oats are the sixth most significant cereal crop in the world, with production exceeding 24 million tons annually, and *Avena sativa* is the most important crop (15). Oats, wheat, barley and rye belong to the same Poaceae family but oats are sub-classified into the Aveneae tribe, while the other cereals belong to the Triticeae tribe. This phylogenetic relationship is exemplified by the homology between oat avenin sequences with those in α- and γ-gliadins of wheat, the B-hordeins of barley and the γ-secalins of rye. Notably, there is no homology with the 33 mer peptide from wheat α-gliadin that encompasses several highly immunostimulatory T cell epitopes in CD. An important distinction is that within the Triticeae tribe, gluten protein makes up 75–80% of the protein in wheat, 45–50% in rye, and 50–55% in barley, but in the Aveneae tribe the equivalent prolamin protein (avenin) makes up only 10–15% of the protein i.e., 1% of the flour (16). This has particular relevance for feeding studies in CD. To illustrate, the consumption of 3–7 g wheat gluten daily induces typical clinical effects in most CD patients after 2 weeks (17), however the equivalent avenin prolamin dose would require the consumption of ~300–700 g oats per day. As a standard serving size is ~30–40 g, this is impractical and makes oat feeding studies aiming to reliably induce and assess biological effects near impossible.

A strict GFD is essential to ensure mucosal healing in CD and a failure to heal is correlated with higher morbidity and mortality (18, 19). This is an onerous, costly and restrictive treatment and its ability to induce CD remission is compromised by poor dietary adherence (20). The GFD is generally lower in dietary fiber and frequently higher in simple carbohydrates and fat (21). The introduction of oats to the GFD increases the range of foods that can be consumed, provides an excellent source of fiber and increases GFD palatability and adherence. Soluble fiber and β-glucan found in oats have been associated with a range of health benefits including reduced serum cholesterol (22, 23). Oats may support the 8–10% of CD patients who also suffer Type 1 diabetes mellitus by lowering post-prandial glycaemia and improving glycaemic control. Long-term oat consumption in CD may also improve quality of life (24).

In the face of these extremely positive nutritional attributes for oats in people with CD yet with the uncertain issue of their safety, there is a strong medical need to resolve this issue. It has become clear from trials in a separate clinical entity, non-celiac wheat sensitivity (NCWS), that properly controlled feeding studies to assess the clinical effects of gluten need to feed purified gluten, and not whole wheat (25). Wheat contains a broad mix of proteins [including gliadins, glutenins, amylase trypsin inhibitors (ATIs), and globulins] and carbohydrates. Recent controlled feeding studies indicate the fermentable carbohydrate component, fructan, is the driver of adverse gastrointestinal symptomatology in many people with NCWS (26). While oats, unlike wheat, is considered low in fermentable carbohydrates (27), it is high in fiber which could trigger adverse gastrointestinal symptoms such as bloating or abdominal discomfort independent of its avenin content. With remarkable foresight, researchers in 1958 reported the challenge of getting children with CD to consume enough oats to establish clinical safety, and identified the need to extract oat protein i.e., avenin to address this challenge (28). At the time, the author lamented, “The possibility of using oat protein itself was explored but although several methods of preparation were attempted, considerable difficulties were encountered.” Thus, there exists a need for purified and uncontaminated food-grade avenin in sufficient quantities for CD feeding studies. We present here a simple method capable of isolating purified, avenin-enriched protein. We demonstrate the avenin is of food-grade standard, uncontaminated by agricultural chemicals and heavy metals, and suitable for human feeding trials.

## Methods

### Protein Content Determination

Protein content was determined by the method of Bradford (29).

### Proximate Composition Analysis

All proximate analyses were completed by a National Association of Testing Authorities accredited food testing facility Agrifood Technology (Werribee, Australia). In brief, protein content was determined using the Kjeldahl method [American Association of Cereal Chemists (AACC) Methods 70-20A and 70-70]; starch and total free sugars (fructose, glucose, lactose, maltose, and sucrose) by in-house developed liquid chromatography—mass spectrometry (LC-MS); β-glucan using enzymatic method after hydrolysis by β-glucosidase (AOAC International Official Method 995.16 and AACC Method 32-23); and water soluble carbohydrates by water extraction and the anthrone method (AFIA 1.11R).

### Urea-SDS-PAGE and Western Blot Analysis

One dimensional urea-sodium dodecyl sulfate polyacrylamide protein gels and Western blots were run as previously described (30). The Sigma-anti-gliadin-horseradish peroxidase (HRP) antibody used here, has been shown to be a general anti-gluten antibody, detecting all gluten protein types present in wheat, barley, rye, and oats (31).

### Oat Cultivation and Purity

Two crops of oats (cv. Wandering, 200 kg) were grown in Williams, in the south-east of Western Australia, using dedicated wheat-free machinery and cropland, and harvested in September 2016 and December 2017. The oats were transported to Melbourne in sealed “bulka” bags and processed in two batches of 200 kg. Prior to grinding of each batch, sequential lots of 100 g of oats was spread thinly on a tray and examined for other grains. No wheat, barley or ryegrass were detected in any samples. No other material was detected, confirming the purity of the oats. The oat grain was ground to pass a 40 hole/in screen, in a dedicated gluten-free hammer mill kindly supplied by Wards Mackenzie (Altona, Australia). The flour was captured in two batches of eight 25 kg bags. Before each bag was sealed, a 100 g flour sample was taken from each bag and screened for accidental contamination with herbicides, pesticides and common aflatoxins (Supplementary Table S1) by Agrifood Technology (Werribee, Australia). Purified avenin was also screened for heavy metal contamination. The flour was extracted using food grade procedures and ethanol in a lab decontaminated to remove traces of wheat flour (Manildra Group, Nowra). All containers used for solvent storage and extraction were Food and Drug Approved and bisphenol A-free (Bunnings, Australia).

### Effect of Solvent Polarity on Small Scale Avenin Precipitation

**Figure 1:**
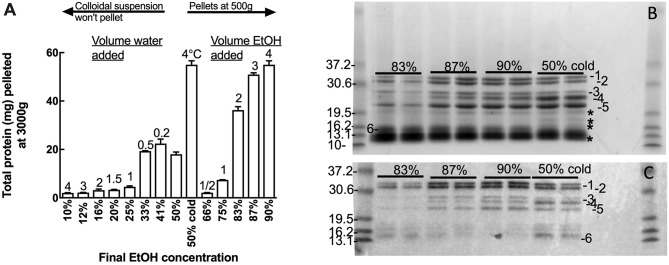
Effect of solvent concentration on avenin precipitation. Adding water or ethanol (EtOH) precipitated avenin, however water-induced precipitate could not be conveniently pelleted. Chill precipitation isolated as much protein as could be isolated by solvent precipitation with a final concentration of 90% ethanol. **(B,C)** Effect of ethanol concentration (83, 87, 90, or 50% chill precipitation) on avenin precipitation by **(B)** protein gel or **(C)** western blot with Sigma anti-gliadin, this antibody has previously been shown to identify all major gluten proteins including avenin (30). Protein bands not corresponding to western bands are indicated on **(B)**, ^*^. Prestained 10 kDa ladder (Benchmark, Invitrogen) is shown to the left of **(B,C)**. The pre-stained standards were themselves calibrated against Benchmark unstained standards which have accurately designated molecular weights.

Oat flour (500 g) was extracted twice in 50% (v/v) ethanol (750 mL) and the extracts pooled. Duplicate 10 mL aliquots of the pooled 50% (v/v) ethanol extract containing 5.2 mg protein/mL was subject to varied total ethanol concentration by diluting the 50% (v/v) ethanol extract with either water, to achieve a final ethanol concentrations of 10–41% (v/v), or with ethanol to achieve final ethanol concentrations of 66–90% (v/v) (Figure 1A). In addition the 50% (v/v) ethanol extract was also chilled at 4°C and centrifuged as below (Figure 1A). Ethanol extracts were centrifuged at 3000 × g for 10 min at room temperature and pellets were dissolved in 8 M urea, 1% (w/v) dithiotreitol (DTT), 20 mM triethylamine-HCl (pH 6) (Urea/DTT) during overnight incubation at room temperature. The protein content of the resultant solutions was measured by Bradford, and 20 and 2 μg protein aliquots were subjected to Urea-SDS-PAGE and western blot respectively (Figures 1B,C).

### Chill Precipitation of a Range of Gluten Proteins

**Figure 2:**
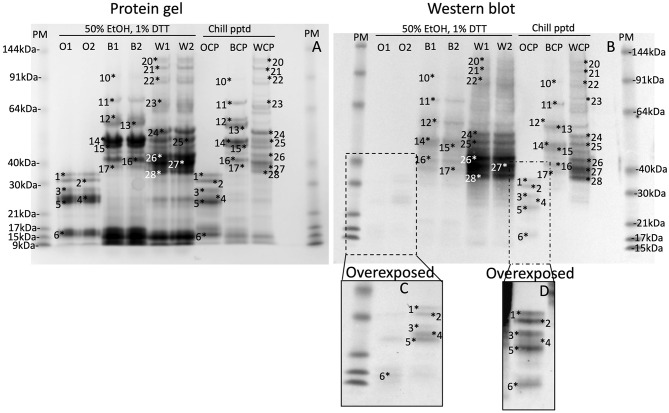
Chill induced precipitation is a general method for all gluten proteins. Gluten proteins were isolated from wheat, barley, and oats by chill precipitation (WCP, BCP, OCP respectively, *Chill pptd*) and compared to gluten proteins freshly isolated by extraction of wheat, barley and oats in 50% EtOH, 1% DTT (W1, W2, B1, B2, O1, and O2, respectively, 50% EtOH, 1% DTT) by SDS-PAGE (**A**; 20 μg per lane), or western blot (**B**; 2 μg protein per lane). Corresponding protein bands, calibrated against pre-stained standards are numbered 1–28 in both images. The inset **(C,D)** shows an overdeveloped section of the western blot to highlight faint bands.

Gluten proteins were isolated from wheat, barley and oat flour by extracting 5 g flour in 15 mL of 50% (v/v) ethanol, vortexing regularly over 1 h, and centrifuging at 3,000x g for 1 h. The 50% ethanol (v/v) supernatants were chilled at 4°C overnight, centrifuged as above and the pellets redissolved in Urea/DTT (wheat 10 mL, barley 10 mL, and oats 1 mL). The prolamin content of the chill precipitates were compared to duplicate extracts of 50 mg (wheat, barley) or 100 mg oats in 1 mL of 50% (v/v) propan-2-ol (IPA), 1% (w/v) DTT (IPA/DTT) which was extracted by violent reciprocal shaking in a Savant bead beater at 30 movements s^−1^ for 1 min and centrifuged at 13,000x g for 5 min. The protein content in the IPA/DTT supernatants and the chill-precipitated pellets were determined and either 20 or 2 μg protein were loaded on each lane of a protein gel and western blot, respectively (Figures 2A,B).

### Avenin Isolation From 500 g Oats

Oat flour (500 g) was shaken regularly over 2 min, 90 min, or for one or 2 days in 750 mL of 50% (v/v) ethanol and then centrifuged at 500 g for 5 min and the supernatant reserved. Pellets were resuspended in 750 mL 50% (v/v) ethanol, re-centrifuged and the process repeated. The yield of protein was determined in the pooled supernatants (Figures S1A,B).

### Large Scale (Sequential) Oat Extraction

Over the course of 9 days in January 2018 and May 2018, two lots of 200 kg of oat flour were ground in a blender to a fine flour, and extracted with 50% (v/v) ethanol as follows. First, 8 kg lots of oat flour were soaked in 12 L of 50% (v/v) ethanol overnight with occasional mixing at room temperature. Tap water was used for all solutions except where noted. In the morning, the oat flour suspension was stirred and decanted into successive 6 × 500 mL buckets and centrifuged at 800 × g for 5 min at 20°C in a Sigma 6-16S centrifuge to give a firm pellet. The clear supernatants were pooled in a 30 L bottle and chilled at 4°C for 1–2 days to selectively precipitate the avenins. The bulk of the avenin settled after 2 days storage at 4°C and was removed from the bottom of the storage container by decanting the supernatant. The avenin precipitate which remained in the supernatant was collected by centrifugation at 5,000x g for 10 min at 4°C and formed a clear honey-like pellet which, with the bulk of the avenin above, was resuspended in a minimum volume of 10% (v/v) ethanol, made with reagent grade 18 MΩ water, and stored at 4°C. Clumps of precipitated avenin were dispersed with an overhead blender, frozen, and freeze-dried in a dedicated gluten-free facility to yield a white powder which was stored dry at −20°C until required. A final yield of 0.9 and 1.2 kg of freeze dried avenin was recovered from each 200 kg oat flour (Figures S2A–C).

### Reversed Phase High Pressure Liquid Chromatography (RP-HPLC) Analysis

Purified, freeze dried avenin (10 mg) was re-solubilized using 70% (v/v) ethanol and vortexed for 30 min (MO BIO Laboratories, Inc. Vortex-Genie^®^ 2). Samples were prepared in triplicate and were centrifuged for 20 min at 15870x g in an Eppendorf Centrifuge 5424. The supernatant was filtered using a 0.45 μm filter. The protein extracts were separated using an Agilent 1200 LC system (Agilent Technologies) using a modified method (32). An aliquot (10 μl) of extract was injected into a C18 reversed-phase Zorbax 300SB-C18 column (4.6 × 150 mm, 5 μm, 300 Å, Agilent Technologies) maintained at 60°C. The eluents used were ultrapure water (solvent A) and acetonitrile (solvent B), each containing 0.1% trifluoroacetic acid (TFA) (HPLC grade, Sigma Aldrich). The separation was carried out using a linear gradient from 33 to 80% solvent B over 65 min at a flow rate of 1 mL/min.

### Protein Profiling Using Matrix-Assisted Laser Desorption Ionization Time-of-Flight Mass Spectrometry (MALDI-TOF-MS) Analysis

Mass spectra representing the protein composition of the prolamin-enriched fraction were obtained from both flour and the purified avenin, and the RP-HPLC fractions collected from the purified avenin sample using MALDI-TOF-MS. Briefly, 60 mg flour sample or 10 mg purified avenin was extracted in triplicate using 300 μL of 70% (v/v) ethanol with vortex mixing for 30 min at room temperature. The protein extract (200 μL) as well as the equivalent amount of eluent from each collected RP-HPLC peak were lyophilized and resuspended in matrix consisting of 40 mg of sinapinic acid (SA), 600 μL of acetonitrile, 360 μL of methanol and 80 μL of water. An aliquot (1 μL) of this matrix was spotted on a 100 spot MALDI-TOF plate and an additional 1 μL sample layer was applied. An Applied Biosystems Voyager DE Pro MALDI-TOF mass spectrometer was operated in linear high mass positive mode using 2050 V laser intensity, an acceleration voltage of 25 kV, grid at 93% and guide wire at 0.2 settings, with 700 ns delay time. A total of 1,000 laser shots were averaged per spectra with three technical replicates analyzed. The detection mass range was set between *m/z* 10,000–60,000.

### LC-MS/MS Analysis of the Collected RP HPLC Peaks

Protein samples were digested by chymotrypsin and peptides were extracted according to standard techniques (33). The peptide samples were analyzed by LC-MS on an Agilent 1260 Infinity HPLC system coupled to an Agilent 1260 Chipcube Nanospray interface on an Agilent 6540 mass spectrometer. Peptides were loaded onto a ProtID-Chip-150 C18 column (Agilent) and separated with a linear gradient of solvent A (2% acetonitrile/97.9% water/0.1% formic acid v/v/v) and solvent B (98% acetonitrile/1.9% water/0.1% formic acid v/v/v) from 2 to 98% solvent B over 18 min. Spectra were analyzed to identify proteins of interest using *in silico* proteolytic digests of Poales subset of UniProt-KB database (accessed 23/05/2019) supplemented with an oat seed transcriptome dataset (34) and appended with the common repository of adventitious proteins (cRAP). The number of protein sequences in the database was 861,955.

### Protein Identification From the Enriched Avenin and Oat Flour

Proteins were extracted in four replicates using the finely ground oat cv Wandering flour and solvents suitable to obtain gluten protein enriched extracts: A −50% (v/v) ethanol; B −55% (v/v) IPA + 2% (w/v) DTT in water following the protocols of Colgrave et al. (31). The purified avenin sample was resolubilized in 50% (v/v) ethanol. From these, 100 μL aliquots of extract were applied to a 10 kDa molecular weight cut-off filter (Millipore, Sydney, Australia), alkylated using iodoacetamide and digested overnight using sequencing-grade trypsin (Promega, Madison, USA) or chymotrypsin (Promega, Madison, USA) as described in Fallahbaghery et al. (35). After digestion, samples were centrifuged at 20,000x g for 15 min and the digested filtrates were dried in a Speedvac (Thermo Fisher Scientific) and the obtained peptides were stored at −20°C until analysis.

Prior to LC-MS analysis the peptides were resuspended in 100 μL of 1% (v/v) formic acid. An aliquot (4 μL) of the peptide digest solutions were separated on an Eksigent NanoLC 415 system (SCIEX, Redwood City, USA) using a trap-elute configuration and 10 μL/min flow rate for trapping and 5 μL/min flow rate for separation. A Protecol C18 120Å trapping column (3 μm particle size, 10 mm × 300 μm, Trajan Scientific, Australia) and an Eksigent ChromXP C18 120Å analytical column (3 μm particle size, 150 mm × 300 μm) was used with a linear gradient from 3 to 25% solvent B over 68 min. Mobile phases consisted of solvent A [0.1% (v/v) formic acid/5% (v/v) dimethyl sulfoxide/94.9% (v/v) water] and solvent B [0.1% (v/v) formic acid/5% (v/v) dimethyl sulfoxide/84.9% (v/v) acetonitrile/10% (v/v) water], and 0.1% (v/v) formic acid/99.9% (v/v) water was used to load the trap column. The eluate was directed into a TripleTOF 6600 MS (SCIEX), operating in information dependent acquisition mode over mass range *m/z* 100–2000. The Paragon algorithm of ProteinPilot 5.0.2 Software (SCIEX) was used for protein identification (36). The tandem mass spectrometry data were searched against the *in silico* proteolytic digests of Poales subset of UniProt database (accessed 23/05/2019) supplemented with the oat seed transcriptome data (34) and appended with the common Repository of Adventitious Proteins (cRAP) database. The database search results were examined and identifications were confirmed if they passed a 1% global false discovery rate (FDR) threshold as determined by the built-in FDR tool within ProteinPilot software (37). The resulting protein dataset was analyzed to identify avenins using the representative prolamin characteristics as described (38). Pfam domains, cysteine residues and major known T cell epitopes (DQ2.5-ave-1a, 1b, 1c, and 2) were mapped to the identified protein sequences using CLC Genomics Workbench v12 (Qiagen, Aarhus Denmark). Protein sequences were aligned using ClustalW algorithm and phylogenetic analysis was performed as reported elsewhere (39).

## Results

### Effect of Solvent Concentration on Avenin Precipitation

Many gluten proteins can be dissolved in 50% (v/v) ethanol or 2-propanol and precipitated by dilution with either water or alcohol. The polarity of the 50% (v/v) ethanol avenin extract was varied by diluting the 50% ethanol extract with water to achieve final concentrations of 10–41% ethanol (v/v), or with ethanol to achieve final concentrations of 66–90% ethanol (v/v) (Figure 1A). All additions (water or ethanol) to the 50% ethanol extract produced a milky white precipitate; however only those precipitates produced by increasing the ethanol addition could be conveniently precipitated. The cloudy precipitate produced by adding water was extremely difficult to spin down and resisted precipitation at 5,000 g, beyond the capacity of the centrifuge used for the large scale avenin preparation. Fortuitously, chilling the 50% ethanol extract at 4°C for 10 min was noted to selectively precipitate avenin, producing a milky white precipitate that could be precipitated at either 500 g or 3000 g over 10 min (Figure S1B) or that settled at 1 g overnight. The chill-induced precipitation of avenin commenced below 15°C and could be reversed by warming, resulting in a clear solution that could be reproduced at least 10 times. The purity of the precipitates produced by varying the ethanol concentration, or chilling, was investigated by comparing proteins (Figure 1B) corresponding with avenin bands on a western blot (Figure 1C). All of the major bands present in the protein gel (Figure 1B, 1–6) correspond to avenins identified in the western blot (Figure 1C, 1-6). Some non-avenin proteins were present in the protein gel (Figure 1B, ^*^). In addition the intensity of both protein and western bands was a maximum in the chill precipitation from 50% ethanol (Figures 1B,C). This indicated that chill precipitation produced an avenin precipitate of high purity showing higher specificity for avenins.

Chilling the water-induced precipitates at 4°C did not help the suspensions to precipitate, possibly due to protein-lipid binding causing the avenins to float. Defatting the oat flour with common defatting solvents (i.e., butanol, ether, or hexane) was not possible due to food safety concerns. For instance, n-hexane biodegrades to form a neurotoxin that must be avoided to maintain food grade standard. Successful and complete defatting of oat flour with 100% ethanol has been reported (40). However, in this study ethanol defatting did not allow protein precipitation at lower centrifugal force, suggesting that the difficulty in pelleting the water-induced precipitates was not due to lipid interaction.

### Chill Precipitation Is a General Method Applicable to a Range of Gluten Proteins

Chill-induced precipitation was assessed for its utility as a general method for isolating gluten proteins. Gluten proteins were isolated from oats, barley and wheat in 50% (v/v) ethanol extracts, followed by chill precipitation (Figures 2A,B, *Chill ppted*, Oats Chill Precipitated (*OCP)*, Barley Chill Precipitated (*BCP)*, Wheat Chill Precipitated (*WCP)*, respectively), and compared to gluten proteins isolated by duplicate extraction of oats, barley and wheat in 50% (v/v) ethanol, 1% (w/v) DTT (Figures 2A,B, *50% ethanol/DTT, O, B, W*, respectively). Previous studies have demonstrated that IPA/DTT is able to efficiently extract the majority of gluten proteins (35). In each case gluten proteins, numbered 1–28 in both images, and by definition extracted by 50% (v/v) ethanol, 1% (w/v) DTT were present in the corresponding 50% (v/v) ethanol chill-precipitated fractions. This was observed for Coomassie stained protein bands (Figure 2A) and gluten-specific proteins by western blot (Figure 2B, highlighted in Figures 2C,D) confirming that chill precipitation isolated the same range of gluten proteins that were isolated from wheat, barley, and oats as extraction in 50% (v/v) ethanol/1% (w/v) DTT, thus confirming the general nature of the chill precipitation method.

### Avenin Yield and Purity

**Figure 3:**
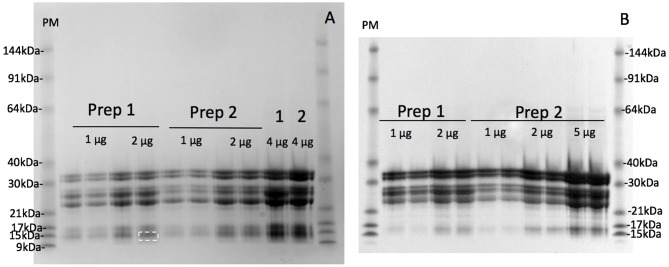
Purity of avenin isolation from 200 kg avenin preparations by SDS-PAGE protein gel **(A)** or western blot **(B)**. Two hundred kilograms of oat flour was successively purified to yield two lots of purified avenin (*Prep 1*) and (*Prep 2*) are shown calibrated against pre-stained 10 kDa ladder (PM). Avenin was dissolved in 8 M urea, 1% DTT, 20 mM triethylamine (pH6), protein measured and 1, 2, 4, or 5 ug loaded per lane as shown. A single non-avenin protein band was present at 14.1 kDa (Figure 2A, *white broken rectangle*) corresponding to 4.2% of total band intensity. This does not correspond to an avenin band in the western blot 2B, and was probably due to α-amylase/trypsin inhibitors. The protein composition of the initial 50% (v/v) ethanol extract of preparation 1 and 2 are shown (**A**, *1* & *2*).

The maximum protein yield was produced by extracting 500 g oat flour in 750 mL of 50% (v/v) ethanol over 1–2 days (Figure S1A). The protein purity of the large scale avenin preparation was firstly examined by resolving protein bands with detection using a general protein stain (Coomassie Blue G250; Figure 3A). The protein bands were then compared to avenin bands identified by a general anti-gluten antibody on a Western blot (Figure 3B) for large scale avenin preparations 1 and 2 (*Prep1* and *Prep2)*. Using calibrated pre-stained standards on the blot and protein gel, avenin bands on both the protein gel and western blot were shown to be of the same molecular weight (Table 1). It is clear that the protein bands (Figure 3A) were due to the dominant avenin bands on the western blot (Figure 3B), except for one protein band at ~14 kDa, which did not correspond to an avenin band in the western blot (Figure 3A, marked by a broken white rectangle). Note that the 9 kDa prestained marker did not bind to the membrane and did not appear in the Western blot (Figure 3B, PM). The protein purity was calculated from the percentage of the protein load attributed to avenin bands in the protein gel. The average purity of both preparations shown for the four 2 μg lanes on the protein gel was 95.8 ± 0.01%.

**Table 1:** Comparison of avenin molecular weights calculated from protein gel and western blots.

**Avenin band**	**Molecular weight (blot) kDa ± SE**	**Molecular weight (gel) kDa ± SE**
1	32.6 ± 0.02	33.0 ± 0.02
2	31.5 ± 0.02	31.5 ± 0.03
3	28.1 ± 0.07	28.6 ± 0.02
4	27.3 ± 0.08	27.3 ± 0.02
5	25.3 ± 0.09	25.3 ± 0.04

*Molecular weights were compared from the protein gel and western blot proteins bands in Figures 3A,B and calibrated against prestained standards*.

### Proximate Composition Analysis

The protein content was assessed by the Kjeldahl method revealing 85.4% protein. The remainder of the preparation consisted of low concentrations of starch, β-glucan, free sugars and water soluble carbohydrates. The starch content was 1.8% on a dry weight (DW) basis. The β-glucan content was 0.2% DW, total free sugars were 1.8% DW and the total water-soluble carbohydrate (WSC) content was 4.6% DW. As starch is insoluble, it does not contribute to the WSC content. The total free sugars (at 1.8% DW) include fructose, glucose, lactose, maltose and sucrose, and are soluble and are thus included in the WSC measurement, leaving 2.8% DW attributed to other sugars, such as oligosaccharides, small fructans, and other complex sugars.

Food grade purity was confirmed by nil detection of herbicides and/or pesticides (Supplementary Table S1). No inadvertent chemical contamination was detected in either of the oat crops. Both oat crops were tested for the presence of aflatoxins (Supplementary Table S1), and all were below the limit of detection (LOD listed in Supplementary Table S1). The purified avenin was also tested for heavy metal contamination. Mercury, chromium and lead were all below the limit of detection. Copper was reported to be 5.1 and aluminum 58 mg/kg. These are within acceptable safety limits. The US Food and Drug Administration (FDA) reports that 10–100 mg aluminum per day is acceptable (41). The proposed feeding trial will involve a daily intake of 0.35 mg aluminum in a 6 g serve of avenin. The FDA does not define a limit on copper safety but these levels and much higher are common in other foods. The Food Standard Australia New Zealand code does not specify levels for copper or aluminum.

### Comparative Proteomic Analysis of the Enriched Avenin and Oat Flour

Previous studies have shown that gluten proteins can be efficiently extracted from gluten using alcohol in the presence of reducing agents. Such solvents are not applicable to production of food grade extracts, but are suitable for biochemical analyses that aim to identify the protein complement in grains, flours, and food products.

Extraction using IPA/DTT showed the highest number of proteins in the oat flour sample after LC-MS/MS analysis of the trypsin digested sample, with 276 proteins identified at the 99% protein confidence level and additional 121 proteins were identified when the confidence level was lowered to 95%. It should be noted that LC-MS/MS is a very sensitive technique capable of detecting proteins that vary in abundance over four orders of magnitude. Detection of a number of non-avenin proteins does not imply reduced purity, but that the technique employed was capable of identifying a range of co-extracted proteins that were not obvious on the gel images (Figure 3). This protein set included 20 avenin sequences, 18 of which were identified with 99% confidence (Table 2). Additionally, there were 25 ATIs, 11 lipid transfer proteins (LTPs) and 15 vromindolines and grain softness proteins identified. A further 38 proteins were identified with the cupin-1 domain that is characteristic of globulins and germins expressed in seeds. The remaining 288 proteins represented a range of enzymes and metabolic proteins. The chymotrypsin digested IPA/DTT extract revealed 136 proteins, of which 106 were detected with 99% protein confidence level. Within this set, 25 avenins (20 with 99% confidence level), 17 ATIs, 5 LTPs, 9 vromindolines and 26 globulins and germins were identified (Table 2). Comparing the trypsin and chymotrypsin digests yielded 10 avenins, 14 ATIs, 7 vromindolines and 4 LTPs that were commonly identified when IPA/DTT extraction was used during the sample preparation.

**Table 2:** Number of detected protein types at 95 and 99% confidence levels.

**Protein type**	**Protein confidence**	**Flour**				**Purified sample**	
**Solvent**		**IPA/DTT**^**a**^		**50% (v/v) ethanol**	
**Enzyme**		**TR^**b**^**	**CTR^**c**^**	**TR**	**CTR**	**TR**	**CTR**
Avenin	99%	18	20	17	19	18	18
	95%	20	25	18	20	21	20
ATI	99%	18	17	15	16	19	16
	95%	24	17	19	16	29	17
Vromindoline and GSP	99%	14	8	10	5	11	6
	95%	15	9	11	5	13	7
LTP and nsLTP	99%	8	4	7	4	6	1
	95%	11	5	9	4	8	3
Globulin-like proteins	99%	32	24	12	8	4	1
	95%	38	26	13	12	6	2
Enzymes and metabolic proteins	99%	186	33	97	25	59	19
	95%	288	54	152	48	103	28
Total number of proteins		397	136	223	105	180	77

a 55% IPA + 2% DTT extraction buffer;

b trypsin;

c *chymotrypsin*.

To obtain comprehensive protein information of the avenin-enriched protein fractions, 50% ethanol extracts of the oat flour and purified avenin were digested using trypsin and chymotrypsin separately, and analyzed. Within the tryptic digests (Table 2), 223 proteins were identified in the flour sample and 180 proteins were identified in the resolubilized avenin sample. Of these, 96 proteins were commonly detected between the flour and the purified avenin extracts. Of the detected proteins in the flour sample 8.1% were avenins, while other prolamin superfamily member proteins were also detected, including ATIs (9%), kernel structure-related vromindoline and grain-softness proteins (4.9%) and LTPs (4.1%). The remaining 164 proteins in the flour extract represented seed storage globulins and proteins with various enzyme and metabolic functions. In the large-scale purified avenin sample, 12% of detected proteins represented avenins, showing a qualitative enrichment in avenin proteins. Of the remaining proteins, 16.6% were ATIs, 7.4% were vromindolines and grain softness proteins and 4.5% were LTPs. The remaining 104 proteins showed a similar composition to that of the flour extract. The chymotryptic peptides yielded 105 protein identifications in the flour sample and 77 proteins in the purified avenin sample with 42 proteins commonly detected between the flour and the purified avenin sample. Identified avenin sequences represented 19% of the detected proteins in the flour sample and 27% in the purified protein sample. Similar to the trypsin digested extracts, ATIs, vromindolines and LTPs were also detected, though at lower numbers than in trypsin digested protein samples (Table 2).

Utilizing chymotrypsin for proteolytic digestion and subsequent LC-MS analysis resulted in an increased number of unique avenin protein identifications independent of the extraction solution used in the analysis. In total, 18% of the identified proteins in the IPA/DTT flour extract were avenins and a similar number (19%) were identified from the 50% ethanol extract of flour. Using trypsin the abundance of avenins within the identified protein pool was significantly lower: 5% for the IPA-DTT and 8% for the 50% ethanol extract (Table 2). Of the trypsin digested avenin proteins 50% were also identified from the chymotrypsin digests. Comparing the number of theoretically detectable tryptic and chymotryptic peptides, a higher number of detectable chymotryptic peptides can be seen in most of the sequences (Supplementary Table S2, Figure S3). This resulted in the detection of an additional 15 avenin proteins in the IPA/DTT and a further 11 avenin sequences in the 50% ethanol extracts when chymotrypsin was used.

Both the oat flour and the purified avenin samples were screened for the presence of wheat and barley contamination using published methods (31, 42) and no evidence was found.

### Protein Molecular Weight and Hydrophobicity Distribution Within the Avenin-Enriched Extract

**Figure 4:**
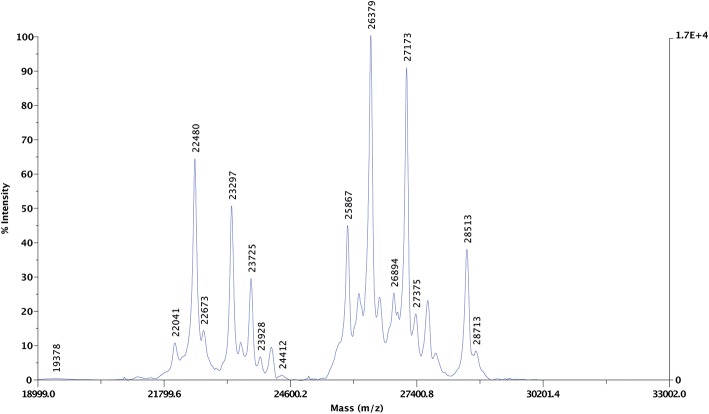
Representative MALDI-TOF MS spectra of the undigested purified protein dissolved in 70% ethanol. The left y-axis represents signal intensity in arbitrary units, the right y-axis shows the maximum intensity value. The x-axis represents the mass-to-charge ratios (*m/z*). The *m/z* values detected from the 70% ethanol resolubilized purified protein extract are highlighted above the peaks.

Altogether 25 protein peaks were identified in the undigested purified extract using MALDI-TOF MS with centroid mass values ranging between 19.3 and 32.4 kDa (Figure 4). The proteins were grouped into three distinct groups: three proteins represented proteins below 22.4 kDa, eight proteins with centroid mass values between 22.4 and 24.4 kDa and 13 proteins with a centroid mass range of 25.8 and 32.4 kDa.

**Figure 5:**
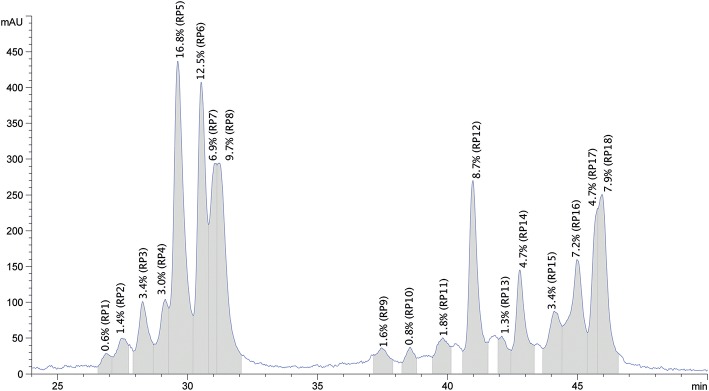
Representative reversed phase HPLC chromatogram of the purified oat protein sample resolubilized in 70% ethanol. The x-axis represents the retention time in min, y-axis shows the relative amount of protein fractions in mAU units. Collected RP peaks and the % peak areas are labeled from RP1 to RP18 and highlighted in gray.

The purified protein sample was also separated based on hydrophobicity using RP-HPLC (Figure 5). The obtained chromatogram peaks were grouped into two retention time ranges. The first group represented peaks between retention time values of 27.0 and 31.3 min and accounted for ~57% of the total protein. In this subgroup peaks between 29.6 and 31.3 min represented the most abundant peaks. The second subgroup that accounted for 43% of the total protein content was composed of 13 peaks and retention time values ranged between 37.6 and 46.0 min. Peaks with retention time values of 41.1, 46.0, and 45.1 min were the most abundant. Based on the obtained chromatogram there were 18 peaks collected and the undigested samples were subjected to protein profiling using MALDI-TOF MS. In parallel to this analysis proteins present in the chymotrypsin-digested RP-HPLC peaks were identified using LC-MS/MS after chymotrypsin digestion.

The RP-HPLC peaks were also quantified by calculating the mean Area (mAU ^*^ s) values of the three replicates × three injections. The absolute amount of protein within each peak was estimated based on the obtained protein content using the Kjeldahl method as described in section Proximate Composition Analysis. The quantitative results, including retention time ranges, calculated mean values as absolute amounts of protein/100 g purified protein are provided in Table 3.

**Table 3:** Identified proteins and their calculated amounts of the collected 18 RP-HPLC peaks.

**RP peak #**	**Retention time peak (min)**	**Collection time range (min)**	**Mean g/100 g protein**	**Range g/100 g protein**
1	26.85	26.6 – 26.9	0.57	0.37–0.72
2	27.38	27.1 – 27.8	1.90	1.65–2.05
3	28.18	28.0 – 28.6	3.29	3.18–3.42
4	29.04	28.7 – 29.1	2.72	2.39–3.04
5	29.54	29.2 – 30.0	17.05	16.64–17.55
6	30.47	30.1 – 30.6	13.13	12.52–13.55
7	30.93	30.7 – 31.0	6.57	6.20–6.89
8	31.15	31.0 – 31.7	10.69	10.10–11.20
9	37.42	37.2 – 37.7	0.99	0.73–1.59
10	38.48	38.2 – 38.6	0.63	0.33–0.85
11	39.72	39.3 – 40.4	1.58	1.40–1.83
12	40.95	40.4 – 41.6	9.29	8.65–9.74
13	41.69	41.9 – 42.3	2.54	1.10–1.28
14	42.77	42.3 – 43.2	5.16	4.50–4.86
15	44.16	43.8 – 44.4	2.99	2.66–3.38
16	44.50	44.4 – 45.3	7.05	6.65–7.53
17	45.94	45.4 – 45.9	4.74	4.56–4.96
18	46.20	46.0 – 46.4	9.11	8.67–9.56

The RP-HPLC peaks RP3-RP7 comprised proteins with the larger molecular mass range (above 26 kDa). Peaks RP1 and RP8 were comprised of proteins spanning the entire mass range while peaks RP9-RP12 mainly included proteins with smaller molecular mass values (below 22.5 kDa). Peaks RP15-RP18 comprised proteins between 22.5 and 24 kDa and above 26 kDa. The number of representative avenin protein types per peak is shown in Table 4. Proteins present in the 18 peaks consisted of avenins and gliadin-like avenins, however peptides characteristic of ATIs were also confidently detected in peak RP2 (Table 4). RP-HPLC analysis indicated that the proteins that eluted between 26.9 and 31.2 min exclusively contained proteins with the DQ2.5-ave-1a epitope and this fraction represented approximately 55.9 g in 100 g purified protein. While DQ2.5 ave-1b epitope containing proteins were detected in the retention time range of 38.5–45.9 min, proteins with DQ2.5-ave-2 epitope were only detected in the RP peak 11.

**Table 4:** Protein characterization of the identified RP-HPLC peaks.

**Fraction #**	No. MS peaks in HPLC fraction^*^	**Observed mass range (m/z)**	**Most intense peaks in MALDI profile**	Accession (Uniprot/ transcript ID)^*^	**Confidence level**	**Monoisotopic mass (signal peptide removed)**	**Epitope**
1	15	20543–28907	28604 27783	L0L6J0 Asat-prolamin54	99% 99%	30789 23496	DQ2.5-ave-1a DQ2.5-ave-1a
2	11	21321–28711	27760 21321 28481	L0L6J0 L0L6K1	99% 95%	30789 22031	DQ2.5-ave-1a DQ2.5-ave-1a
3	9	21327–28652	28467	L0L6J0	99%	30789	DQ2.5-ave-1a
4	6	22038–28675	28498	Asat-prolamin2 Q09072	99% 95%	24703 23539	DQ2.5-ave-1a DQ2.5-ave-1a
5	5	22049–28704	28486	Asat-Prolamin10 L0L6K1	99% 95%	25189 22031	DQ2.5-ave-1a
6	4	27609–28659	28478	Asat-Prolamin54 L0L6K1 Asat-Prolamin10	99% 99% 99%	23496 22031 25189	DQ2.5-ave-1a DQ2.5-ave-1a DQ2.5-ave-1a
7	4	22009–28481	27595	L0L6J0 L0L6K1	99% 99%	30789 22031	DQ2.5-ave-1a DQ2.5-ave-1a
8	10	21240–28516	24184 22040 27652	Asat-Prolamin10 Q09072	99% 99%	25189 23539	DQ2.5-ave-1a DQ2.5-ave-1a
9	2	22513–22711	22513	L0L4J7	99%	22481	DQ2.5-ave-1c
10	4	21893–25833	22465	L0L4J7 L0L6J0	99% 99%	22481 30789	DQ2.5-ave-1c DQ2.5-ave-1a
11	2	21792–22495	22495	L0L4J7 Q09072 I4EP88 I4EP86 L0L6J0	99% 99% 99% 95% 95%	22481 23539 25862 21915 30789	DQ2.5-ave-1c DQ2.5-ave-1a DQ2.5-ave-1b DQ2.5-ave-2 DQ2.5-ave-1a
12	3	22495–22703	22495 22598	L0L4J7 I4EP58	99% 95%	22481 27966	DQ2.5-ave-1c DQ2.5-ave-1b
13	5	22481–25855	22481	L0L4J7 Asat-Prolamin71 L0L6J0	99% 99% 95%	22481 22752 30789	DQ2.5-ave-1c DQ2.5-ave-1b DQ2.5-ave-1a
14	5	22474–26051	25850	I4EP58	99%	27966	DQ2.5-ave-1b
15	11	22642–27149	25853 23314	Asat-Prolamin71 G8ZCU7 Q09097	99% 99% 99%	22752 21628 Fragment	DQ2.5-ave-1b DQ2.5-ave-1c
16	7	23317–27187	23735	I4EP54 L0L6J0 Asat-Prolamin15	99% 99% 95%	26895 30789 20556	DQ2.5-ave-1b DQ2.5-ave-1a DQ2.5-ave-1c
17	10	23317–27328	23729	I4EP58 Asat-Prolamin15	99% 99%	27966 20556	DQ2.5-ave-1b DQ2.5-ave-1c
18	8	23294–27363	23294 26370	I4EP57	99%	27948	DQ2.5-ave-1b

* *Peaks above 10% intensity in MALDI-TOF MS were considered*.

### Characterization of the Identified Avenin Types

**Figure 6:**
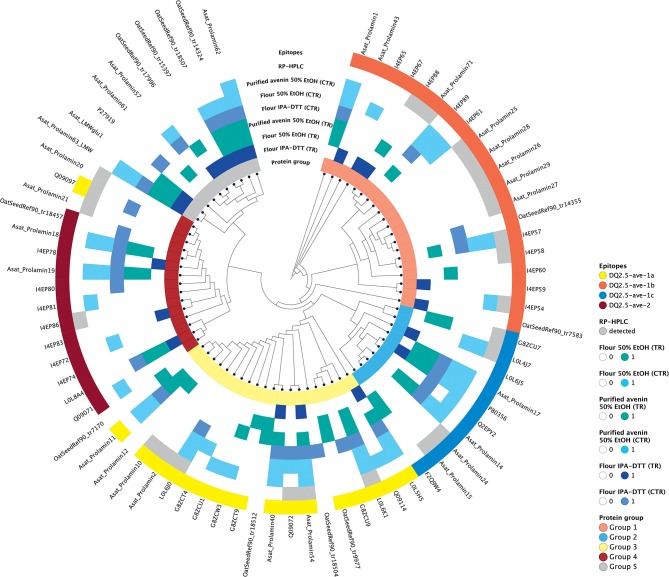
Epitope mapping and phylogenetic analysis of avenin sequences identified from the different protein extracts. Avenin sequences identified from any of the analyses are shown in the tree and were grouped into five groups (labeled in the inner circle). The sequences that contain epitopes DQ2.5-ave-1a, DQ2.5-ave-1b, DQ2.5-ave-1c, and DQ2.5-ave-2 are highlighted yellow, red, blue, and dark red, respectively, in the outer ring. Sequences within the same phylogenetic groups without these epitopes are shown as empty blocks. Those proteins identified using the different protein extraction methods and enzymes are labeled with blue and teal colored blocks in intermediate rings. Proteins identified from the individual RP HPLC peaks are labeled in gray in the outer ring.

As the purpose of the avenin feeding trials is to provoke a T cell response to assess the clinical significance of oats in CD, it was important for us to confirm the presence of the previously reported and potentially pathogenic avenin peptides that encompass T cell epitopes (13). Avenin protein sequences that were detected in any of the analyzed samples were used for phylogenetic analysis and epitope mapping. The identity of the known T cell epitopes and the protein identification analyses of oat flour and purified avenin samples along with RP peak analysis were used to annotate the phylogenetic tree and compare the characteristics of the sequence groups. The sequences were grouped into major clusters according to the presence of conserved avenin-specific T cell epitopes (Figure 6, Supplementary Table S2). Group 1 included proteins with the epitope DQ2.5-ave-1b, the majority of the group 2 sequences contained DQ2.5-ave-1c, group 3, the largest group, was typified by the presence of DQ2.5-ave-1a and group 4 was typified by the presence of DQ2.5-ave-2. The sub-branch without T cell epitopes (group 5) represents protein sequences mostly similar to high and low molecular weight glutenin-like sequences. The results clearly indicate that the 50% ethanol extraction method is suitable to extract the major epitope containing avenin types. The resultant purified protein contains all of the reported immunogenic avenin peptides, and therefore should be suitable as a way to assess their clinical toxicity in feeding studies.

## Discussion

Determining if oats should be excluded or included in the GFD is an important issue with medical and societal implications. Dedicated, controlled feeding studies are needed to definitively resolve this issue. Here, we demonstrate an approach that enables the production of a highly purified oat avenin preparation, containing all the known immunogenic avenin T cell epitopes, which contains minimal levels of other carbohydrates or proteins which could otherwise confound the interpretation of the feeding trial. This avenin preparation will facilitate immune and feeding studies to test the suitability of oats in CD. Studies can now be undertaken to assess the immunogenicity of this protein using patient-derived gluten-specific T cells, and employ feeding studies in patients with CD to determine its biological effects.

Despite the many nutritional benefits of oats, contradictory clinical feeding studies, and lack of a clear scientific rationale for dietary guidelines have led to differing views on the safety of oats consumption in CD. A systematic review on studies of oat safety in CD (4) identified several limitations including (i) small sample sizes: uncommon patients with oats sensitivity may not have been included; (ii) assessment of GFD adherence: often not reported; (iii) adverse symptoms: cannot determine if they are related to oats avenin or the fiber load of oats itself; and (vi) oat cultivars: usually not reported. Furthermore, study withdrawals which were often due to adverse gastrointestinal symptoms and/or the inability to maintain the oats diet, may have underestimated the adverse impact of oats intake, and recruitment bias, may lead to oats-sensitive patients avoiding participating in oats feeding studies. The authors concluded: “Our confidence is limited by the low quality and limited geographic distribution of the data” and “Rigorous double-blind, placebo-controlled, randomized controlled trials, using commonly available oats sourced from different regions, are needed.” (4).

An important consideration is that testing for cereal contamination over the last two decades has highlighted a high frequency of commercial oats brands contaminated by wheat, barley and/or rye prolamin in amounts toxic to CD patients (43–45). Contamination can occur since wheat, barley, and oats are grown in the same areas and often harvested and transported with the same machinery. The presence of a single grain of wheat in 200 g of oats can result in the wheat gluten level of >100 ppm; well above the 20 ppm level set in most legislations as the upper limit for gluten-free food status. Failure to provide harvesting, transport and milling facilities dedicated to oats may easily result in significant inadvertent contamination by wheat grains.

Another challenge with oat feeding studies may arise from supplying insufficient avenin to provoke a measurable response in people with CD. With a short-term oral oat challenge of 100 g daily, 8% of patients with CD had pro-inflammatory T cell responses detectable in the bloodstream (13) in contrast to the 75–80% that would be seen after just four slices of wheat bread daily (46). To fully assess the clinical safety of oats we argue it will be important to deliver oats avenin at a “dose” sufficient to trigger both immune and biologic responses. While immune studies suggest oats avenin contain immunogenic sequences capable of stimulating gluten-specific T cells in CD, such a study may help establish a safe threshold dose of oats for consumption in CD by correlating these findings with clinically important endpoints.

Here, we demonstrate that it is possible to produce 85% pure oat grain storage protein extracts from oat flour using repeated 8 kg scale avenin-enriched extractions. The IPA/DTT method combined with trypsin digestion yields a large number of gluten proteins from cereal grains such as wheat (31). This analysis in oats demonstrated that using 50% (v/v) ethanol was sufficient to identify a majority of the avenin protein types. The least represented group was that of proteins with the DQ2.5-ave-1b epitope. This protein type however, was extracted in higher number using the IPA/DTT solvent. Similarly, when reducing agent was added during the purified avenin resolubilization the protein group was better represented (data not shown). This indicates that these proteins with 9 cysteine residues in their sequence might be part of the protein polymer and therefore less soluble in the absence of reducing agent (Supplementary Table S2).

The final avenin preparation contains the avenin bands present in the initial crude 50% (v/v) ethanol extract. Thus, the majority of avenins are recovered by this process. Ninety six percent of the protein in the final protein preparation was due to authentic avenins which were identified by western blotting. The content of starch, β-glucan, sugars, and fatty acids were low in the final avenin preparation. Using repeated extractions it is possible to process 200 kg of oat flour in 9 days to produce ~1 kg of food-grade, avenin-enriched freeze-dried powder.

The RP-HPLC peak analysis indicated the presence of ATIs in the purified sample and this was verified with the comparative analysis using various extraction methods. The number of ATIs detected in the 50% (v/v) ethanol purified protein was in the same range as the avenins. Western blots show the presence of ATI is <4.2% however the precise amount requires further analysis. Other prolamin proteins were also detected. The number of LTPs and kernel structure-related vromindolines was in the same range as of the avenins. Furthermore, globulins, enzymes, and metabolic proteins were also present in large number. Comparison of the IPA/DTT extracted flour sample and the protein preparate clearly shows the depletion of other protein types in the protein preparate and the increase of detected avenin sequences. Using RP-HPLC analysis combined with the MALDI-TOF MS and LC-MS confirmed the presence of significant amount of avenins in the sample. RP-HPLC peaks 5, 6, 8 altogether represent about 40% of the measured protein amount. These peaks were primarily enriched in DQ2.5-ave-1a containing avenins.

Although CD is not a food allergy, controlling for potential confounding effects from ATIs or LTPs in the subsequent analysis of the avenin preparation will be important as these proteins have been associated with human disease including food allergies and Baker's asthma. ATIs have also been implicated in *in vitro* studies to contribute to intestinal inflammation via activation of innate immune pathways (47, 48). Utilizing readouts specific to CD and gluten such as gluten-specific T cells will ensure assessment is focussed on biological effects driven by the avenin and not other components.

The public databases are relatively poor in oat specific storage protein sequences, therefore wild relatives of *Avenae* were also included in the analysis. In total, 185 avenin or avenin-like gliadin sequences were included in the data background. Due to this poor avenin protein sequence representation a precise protein identification is rather challenging but our results clearly indicated that the purified protein is enriched in avenin and gliadin-like proteins. Importantly, using an expanded prolamin database that also includes prolamin sequences of other Poales species, including wheat and barley, we have excluded the possibility of wheat and barley contamination in the oat purified protein.

Comparison of the MALDI-TOF and MicroLC-TripleTOF MS analysis results also revealed that, similar to wheat gliadins, the avenins show a significant level of genetic variability, indicating the presence of multiple gene copies with largely similar protein size and hydrophobicity within the used cultivar. It also indicates the possible presence of avenin alleles with slightly different protein mass values in the different *Avena sativa* cultivars and *Avena* species. Some of the protein peaks were present in multiple adjacent RP-HPLC peaks which might be due to the resolution limitations of RP-HPLC. Mass differences <100 Da observed in adjacent RP peaks can be explained by the presence of post-translational modifications and highly similar sequences with amino acid substitutions or insertions/deletions. Although the overlapping protein set between the different extraction protocols and the RP HPLC peak analysis was small, the avenin sequence analysis confirmed that proteins from the same avenin sub-types were detected. The background database used included <200 avenin sequences representing a rather narrow genetic variability of only a few *Avena* species and *Avena sativa* cultivars. The avenin sequences within the analysis set share 70–99% sequence identity within the groups and 24–45% sequence identity between the groups. Only a few avenin proteins were identified with nearly complete peptide coverage. This also demonstrates that the avenin sequences present in the cultivar Wandering are different from those in the public databases.

## Conclusion

Resolving the issue of oat safety in people with CD will require feeding studies to assess the clinical and immune effects of pure oat avenin. The purity of oat avenin can be confirmed by rigorous proteomic characterization to control for confounding factors such as contamination or potential cross reactivity. A requirement for purified oat avenin for feeding studies was identified as far back as the 1950's, but until recently, production of this at a scale and purity suitable for human feeding studies was not possible. With the method reported here, it is possible for the first time to generate highly pure avenin suitable for controlled feedings studies in CD. This will allow the biological effects of oats avenin to be assessed devoid of the confounding effects from other oat proteins, sugars or fiber, or non-oat sources of gluten. We believe this is a crucial advance that will allow this issue to be definitively assessed and resolved.

## Data Availability Statement

All datasets generated for this study are included in the manuscript/Supplementary Files.

## Author Contributions

JT-D conceived the study. The method was developed by GT and FB. The large-scale purification was completed by GT and FB. Protein analysis was completed by GT. RP-HPLC and MALDI-TOF analysis was carried out by CF and quantitatively evaluated by FB. Manuscript analysis and proteomic data analysis was carried out by AJ, MN-W, and MC. Bioinformatic analysis was completed by AJ. Proximate analysis was supervised by AR. MH contributed to study design and epitope analysis. All authors contributed to the writing of the manuscript.

### Conflict of Interest

GT and JT-D are co-authors on a patent to use chill precipitation for gluten isolation (Provisional Patent Application no. 16/291,326) and Australia (Patent Application No. 2019201465). JT-D and MH are co-inventors of patents pertaining to the use of gluten peptides in therapeutics, diagnostics, and non-toxic gluten in celiac disease. JT-D holds shares in Nexpep Pty. Ltd. and is on the Scientific Advisory Board of ImmusanT, Inc. The remaining authors declare that the research was conducted in the absence of any commercial or financial relationships that could be construed as a potential conflict of interest.

## Abbreviations

AACC: American Association of Cereal Chemists
ATIs: α-amylase/trypsin inhibitors
CD: coeliac disease
cRAP: common Repository of Adventitious Proteins
DTT: dithiothreitol
FDA: US Food and Drug Administration
GFD: gluten-free diet
HRP: horseradish peroxidase
IPA: propan-2-ol
IPA/DTT: 50% (v/v) IPA, 1% (w/v) DTT
LC-MS/MS: liquid chromatography tandem mass spectrometry
LOD: limit of detection
MALDI-TOF-MS: matrix-assisted laser desorption ionization time-of-flight mass spectrometry
NCWS: non-celiac wheat sensitivity
RP-HPLC: reverse phase high pressure liquid chromatography
Urea/DTT: 8 M urea, 1% (w/v) DTT, 20 mM triethylamine-HCl (pH 6).
