# Supplementary material for: Preparation and Characterization of Avenin-Enriched Oat Protein by Chill Precipitation for Feeding Trials in Celiac Disease
Source: Front Nutr. 2019 Oct 15;6:162. doi: 10.3389/fnut.2019.00162 (PMC6803533; doi:10.3389/fnut.2019.00162)
Supplement: Supplementary file 1 [file Data_Sheet_1.docx]

**Supplementary Table S1.** **Screening analysis for herbicides, pesticides, mycotoxins and metals carried out by Agrifood Technology (Werribee, Australia)**

| **AT3 Chemicals**  **(LOD <0.010 (mg/kg)** | | **AT5 chemicals**  **(LOD <0.010 (mg/kg)** |
| --- | --- | --- |
| 2-phenylphenol | Fenthion | Azoxystrobin |
| Abamectin | Fenvalerate | Boscalid |
| Acephate | Fenvalerate (Total) | Bromacil |
| Aldrin | Fipronil | Carbendazim / Benomyl |
| Atrazine | Fludioxonil | Chlorantraniliprole |
| Azinphos methyl | Flusilazole | Dithianon |
| Benalaxyl | Fluvalinate | Diuron |
| BHC alpha | Fluvalinate tau | Fenhexamid |
| BHC beta | HCB | Fenpyroximate |
| BHC delta | Heptachlor | Flubendiamide |
| BHC gamma (Lindane) | Heptachlor epoxide | Imidacloprid |
| BHC Total | Hexaconazole | Methomyl |
| Bifenazate | Hexythiazox | Methomyl Oxime |
| Bifenthrin | Imazalil | Pymetrozine |
| Bioresmethrin | Indoxacarb | Pyraclostrobin |
| Bitertanol | Iprodione | Spinetoram |
| Buprofezin | Kresoxim methyl | Spinosad |
| Captan | Linuron | Spirotetramat |
| Carbaryl | Malathion | Tebufenozide |
| Chlordane | Metalaxyl | Thiabendazole |
| Chlorfenapyr | Methamidophos | Thiacloprid |
| Chlorfenvinphos | Methidathion | Thiamethoxam |
| Chlorothalonil | Metribuzin | Trifloxystrobin |
| Chlorpyrifos | Mevinphos |  |
| Chlorpyrifos methyl | Monocrotophos |  |
| Chlorthal dimethyl | Myclobutanil |  |
| Clofentezine | Omethoate |  |
| Cyfluthrin | Oxyfluorfen | **LCMS - MS Mycotoxin Screen**  **(LOD as shown)** |
| Cyfluthrin beta | Paclobutrazol | Aflatoxin B1 < 1.0 µg/kg |
| Cyhalothrin lambda | Parathion ethyl | Aflatoxin B2 < 1.0 µg/kg |
| Cypermethrin | Parathion methyl | Aflatoxin G1 < 1.0 µg/kg |
| Cypermethrin alpha | Penconazole | Aflatoxin G2 < 1.0 µg/kg |
| Cyproconazole | Pendimethalin | Deoxynivalenol < 100µg/kg |
| Cyprodinil | Penthiopyrad | Fumonisin B1 < 20 µg/kg |
| DDD p,p | Permethrin | Fumonisin B2 < 20 µg/kg |
| DDE p,p | Phenothrin | HT2 < 20 µg/kg |
| DDT p,p | Phorate | Nivalenol < 100µg/kg |
| DDT Total | Phosmet | Ochratoxin A < 1.0 µg/kg |
| Deltamethrin | Piperonyl butoxide | T2 < 1.0 µg/kg |
| Diazinon | Pirimicarb | Total Aflatoxin (B1, B2, G1, G2)  < 4.0 µg/kg |
| Dichlorvos | Pirimiphos methyl | Zearelenoneb < 1.0 µg/kg |
| Dicloran | Prochloraz |  |
| Dicofol | Procymidone |  |
| Dieldrin | Profenofos |  |
| Difenoconazole | Propargite |  |
| Dimethoate | Propiconazole | **Mercury** |
| Dimethoate (Total) | Prothiofos | Mercury < 1.0 µg/kg |
| Dimethomorph | Pyrethrins |  |
| Diphenylamine | Pyrimethanil |  |
| Disulfoton | Pyriproxyfen |  |
| Endosulphan alpha | Quintozene |  |
| Endosulphan beta | Sulfoxaflor | **Metals** |
| Endosulphan sulphate | Tebuconazole | Aluminium† 58 mg/kg |
| Endosulphan Total | Tebufenpyrad | Chromium < 0.010 mg/kg |
| Endrin Total | Terbufos | Copper 5.1 mg/kg |
| Esfenvalerate | Tetradifon | Lead < 0.030 mg/kg |
| Ethoprofos | Tolclofos methyl |  |
| Etoxazole | Triadimefon |  |
| Fenamiphos | Triadimenol | † Not NATA accredited |
| Fenarimol | Trichlorfon |  |
| Fenitrothion | Vinclozolin |  |
| Fenoxycarb |  |  |


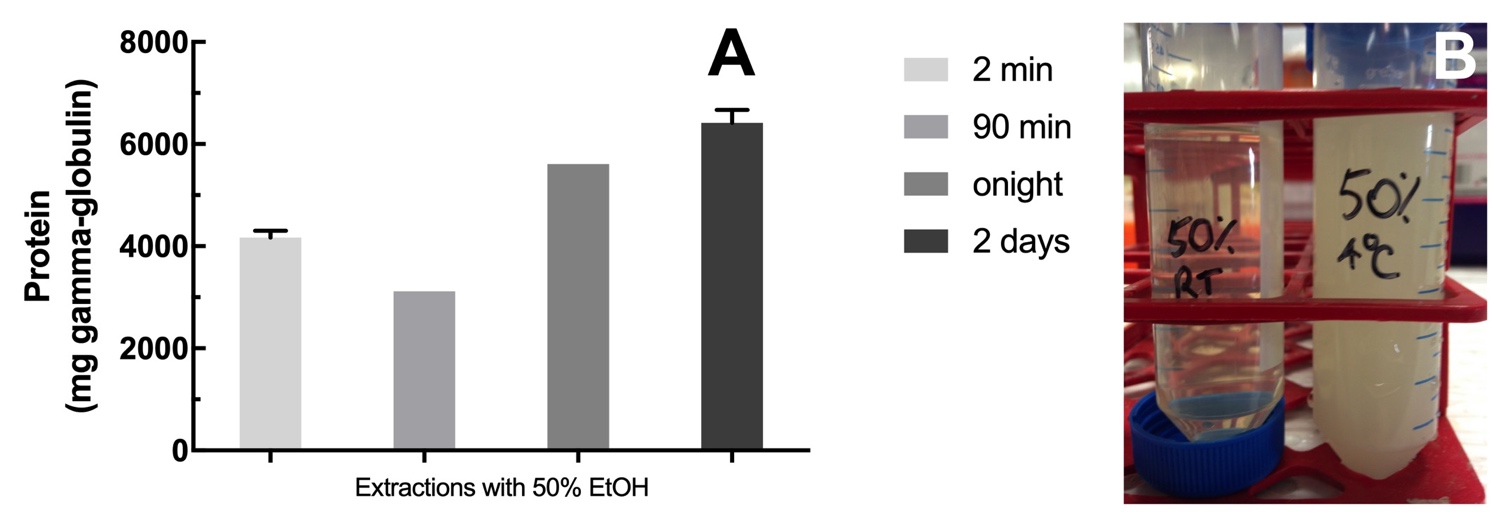


**Figure S1. (A)** The effect of extraction on avenin yield. Maximum freeze-dried avenin yield was observed after a two-day extraction. Oat flour (500 g) was extracted with 50% EtOH v/v for the indicated time and protein content of the supernatant measured. (**B**) Avenin precipitation by chilling at 4°C. A precipitate formed as the 50% EtOH extract cooled below 15°C and appeared to be complete at 10°C. This could be reversed 10 times, by warming to 20°C and then cooling again to 4°C.


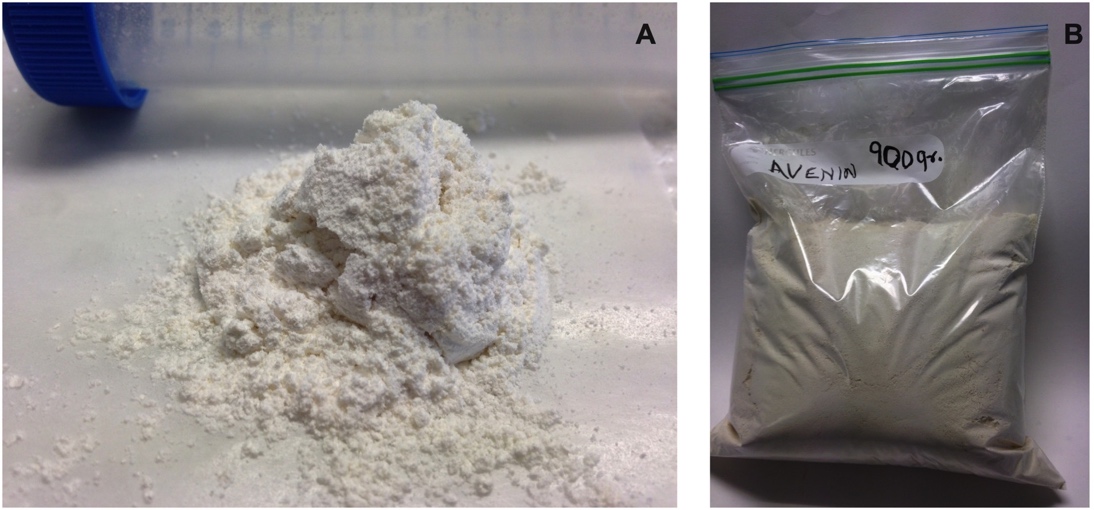


**
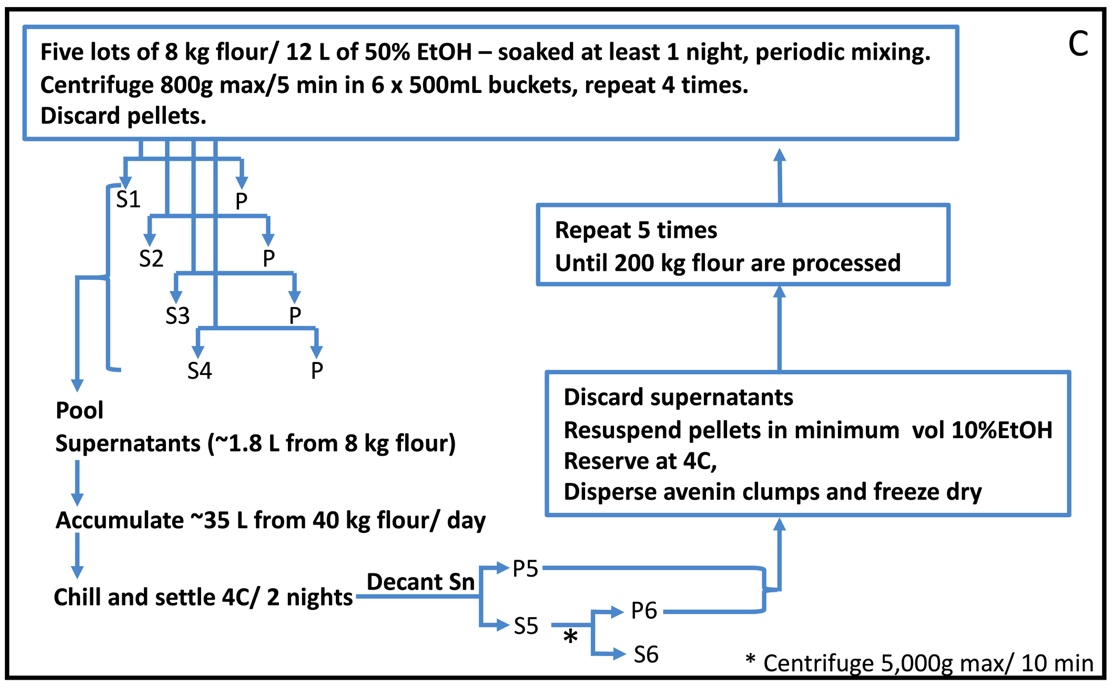
**

**Figure S2.** Results of large scale avenin isolation by chill precipitation showing: **(A**) 10 g fine powder; (**B**) 900 g of freeze dried purified avenin preparation, and (**C**) repeated large scale preparation protocol for avenin isolation.

**
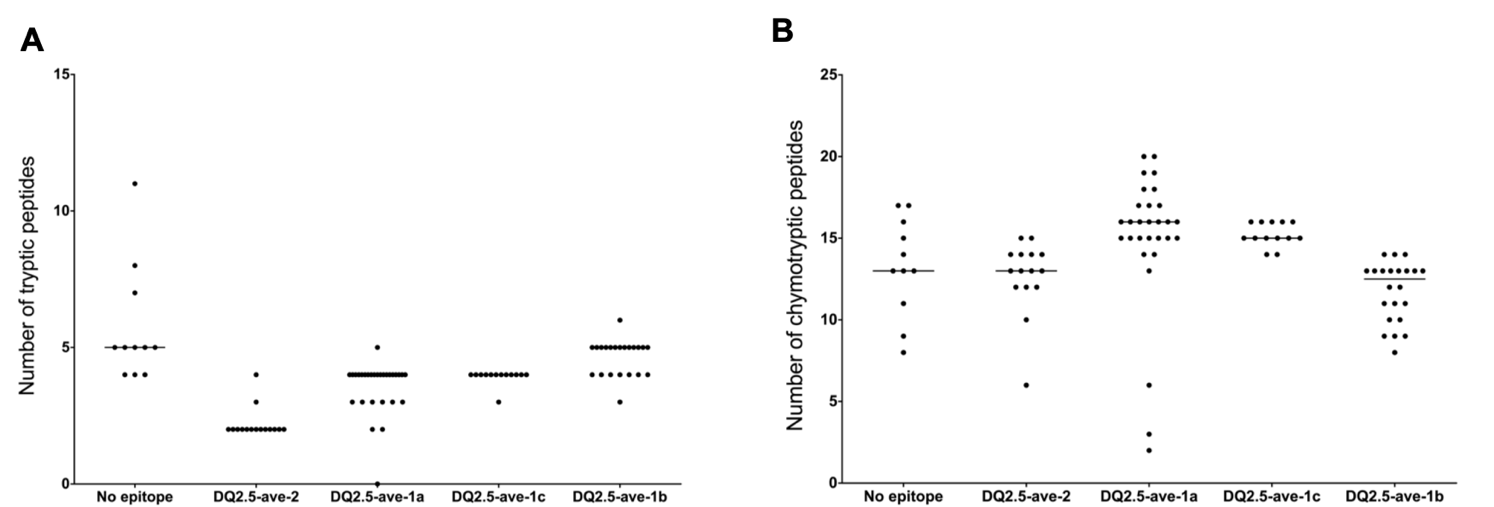
**

**Figure S3**. Comparison of detectable avenin peptides obtained using trypsin **(A)** and chymotrypsin **(B)** digestion *in silico*. Number of peptides and the median values are shown for each epitope group individually.
